# Impact of ^18^F-FET PET/MRI on Clinical Management of Brain Tumor Patients

**DOI:** 10.2967/jnumed.121.262051

**Published:** 2022-04

**Authors:** Cornelia Brendle, Caroline Maier, Benjamin Bender, Jens Schittenhelm, Frank Paulsen, Mirjam Renovanz, Constantin Roder, Salvador Castaneda-Vega, Ghazaleh Tabatabai, Ulrike Ernemann, Christian la Fougère

**Affiliations:** 1 Diagnostic and Interventional Neuroradiology, Department of Radiology, University Hospital Tuebingen, Tuebingen, Germany;; 2 Diagnostic and Interventional Radiology, Department of Radiology, University Hospital Tuebingen, Tuebingen, Germany;; 3 Department of Radiology, Cantonal Hospital Muensterlingen, Muensterlingen, Switzerland;; 4 Department of Neuropathology, University Hospital Tübingen, Tuebingen, Germany;; 5 Department of Radiation Oncology, University Hospital Tuebingen, Tuebingen, Germany;; 6 Department of Neurology and Neurooncology, University Hospital Tuebingen, Hertie Institute for Clinical Brain Research, Tübingen, Germany;; 7 Department of Neurosurgery, University Hospital Tübingen, Tuebingen, Germany;; 8 Nuclear Medicine and Clinical Molecular Imaging, Department of Radiology, University Hospital Tuebingen, Tuebingen, Germany;; 9 Werner Siemens Imaging Center, Department of Preclinical Imaging and Radiopharmacy, Eberhard Karls University Tuebingen, and University Medical Center, Tuebingen, Germany;; 10 Cluster of Excellence iFIT (EXC 2180) “Image Guided and Functionally Instructed Tumor Therapies,” University of Tuebingen, Tuebingen, Germany; and; 11 German Cancer Consortium, Partner Site Tuebingen, Tuebingen, Germany

**Keywords:** multiparametric ^18^F-FET PET/MRI, brain tumor, accuracy, clinical impact, human

## Abstract

Multiparametric PET/MRI with the amino-acid analog *O*-(2-^18^F-fluoroethyl)-l-tyrosine (^18^F-FET) enables the simultaneous assessment of molecular, morphologic, and functional brain tumor characteristics. Although it is considered the most accurate noninvasive approach in brain tumors, its relevance for patient management is still under debate. Here, we report the diagnostic performance of ^18^F-FET PET/MRI and its impact on clinical management in a retrospective patient cohort. **Methods:** We retrospectively analyzed brain tumor patients who underwent ^18^F-FET PET/MRI between 2017 and 2018. ^18^F-FET PET/MRI examinations were indicated clinically because of equivocal standard imaging results or the clinical course. Histologic confirmation or clinical and standard imaging follow-up served as the reference standard. We evaluated ^18^F-FET PET/MRI accuracy in identifying malignancy in untreated suspected lesions (category, new diagnosis) and true progression during adjuvant treatment (category, detection of progression) in a clinical setting. Using multiple regression, we also estimated the contribution of single modalities to produce an optimal PET/MRI outcome. We assessed the recommended and applied therapies before and after ^18^F-FET PET/MRI and noted whether the treatment changed on the basis of the ^18^F-FET PET/MRI outcome. **Results:** We included 189 patients in the study. ^18^F-FET PET/MRI allowed the identification of malignancy at new diagnosis with an accuracy of 85% and identified true progression with an accuracy of 93%. Contrast enhancement, ^18^F-FET PET uptake, and tracer kinetics were the major contributors to an optimal PET/MRI outcome. In the previously equivocal patients, ^18^F-FET PET/MRI changed the clinical management in 33% of the untreated lesions and 53% of the cases of tumor progression. **Conclusion:** Our results suggest that ^18^F-FET PET/MRI helps clarify equivocal conditions and profoundly supports the clinical management of brain tumor patients. The optimal modality setting for ^18^F-FET PET/MRI and the clinical value of a simultaneous examination need further exploration. At a new diagnosis, multiparametric ^18^F-FET PET/MRI might help prevent unnecessary invasive procedures by ruling out malignancy; however, adding static ^18^F-FET PET to an already existing MRI examination seems to be of equal value. At detection of progression, multiparametric ^18^F-FET PET/MRI may increase therapy effectiveness by distinguishing between tumor progression and therapy-related imaging alterations.

## Visual Abstract

**Figure:**
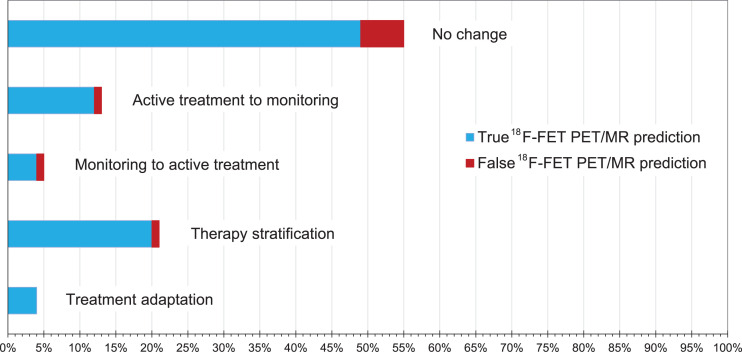

PET with radiolabeled amino-acid analogs such as *O*-(2-^18^F-fluoroethyl)-l-tyrosine (^18^F-FET) is an advanced noninvasive imaging method for various disease-related indications of brain tumors (*1*–*5*). Combining it with MRI using a hybrid scanner may further improve its diagnostic validity (*6**,**7*). However, excellent diagnostic performance does not necessarily correlate with better patient outcomes. To determine a diagnostic procedure’s actual clinical utility, one must additionally assess its impact on clinical management, patient-relevant outcomes, and cost-effectiveness (*8**,**9*).

There is limited evidence of the impact of PET on clinical decisions (*10*). Single studies have reported clinical management changes in a significant proportion of patients (*11*–*14*). Ideally, scientific studies should compare the clinical consequence of a new procedure with an established diagnostic test in a randomized, controlled design (*9**,**10*). This comparison can be challenging for several reasons. First, the patient outcome depends mainly on applied therapies. Extensive sample sizes will be needed to filter out a diagnostic procedure’s small and multifold impact on a patient cohort with various therapeutic approaches (*15*–*17*). Also, an artificial patient selection may not reflect the disease’s actual prevalence and distribution of clinical manifestations (*15*). Amino acid PET/MRI in brain tumors currently serves as add-on diagnostics in patients with equivocal findings on clinical routine MRI. A direct comparison of both modalities would not be helpful. Therefore, several authors recommend performing studies in a routine clinical setup (*15**,**16*).

Summarized, defining the impact of an imaging procedure such as PET/MRI is challenging but essential to establish an efficient application in clinical routine. We aimed to investigate the clinical consequences of multiparametric ^18^F-FET PET/MRI in brain tumors by performing a structured evaluation of its diagnostic performance and impact on clinical management under real-world conditions.

## MATERIALS AND METHODS

### Patients and Data Collection

The institutional review board approved this retrospective study, and all subjects gave written informed consent. We retrospectively reviewed all ^18^F-FET PET/MRI brain tumor examinations and disease outcomes in our institution in 2017 and 2018. Our institution treats over 600 newly diagnosed brain tumor patients per year. ^18^F-FET PET/MRI serves as a second-line diagnostic procedure performed only on recommendation by a multidisciplinary tumor board in a minority of cases at both initial diagnosis and during the disease course. Thus, ^18^F-FET PET/MRI is performed predominately in patients presenting with uncertain MRI features or an equivocal clinical course after or during treatment. We evaluated all clinical data from the patient reports of each medical specialty and the multidisciplinary neurooncologic tumor board. We recorded the patient age and sex, tumor pathology, periods between examinations, and follow-up duration. We also documented the medical history, including treatment recommendations immediately before the ^18^F-FET PET/MRI examination, and the subsequent disease course, including pathologic examinations, subsequent therapies, clinical status, and imaging follow-up. In single cases, we could not retrieve retrospectively precise information about the treatment recommendations before ^18^F-FET PET/MRI. The clinical specialists of the tumor board reviewed these cases for the study and determined the appropriate treatment recommendation.

### ^18^F-FET PET/MRI Examinations and Data Analysis

All ^18^F-FET PET/MRI examinations were performed on a hybrid 3-T PET/MRI scanner (Biograph mMR; Siemens Healthineers) for the clinical indication. An ultrashort-echo-time MRI sequence provided by the vendor was used for PET attenuation correction. The diagnostic MRI comprised sequences according to the standardized brain tumor protocol, dynamic susceptibility contrast perfusion MRI, and ^1^H-MR spectroscopy (MRS) (*18*–*20*). Multislice dynamic susceptibility contrast perfusion MRI was assessed during the first pass of a 0.1 mmol/kg bolus of gadobutrol (Gadovist [Bayer Healthcare]; injection rate of 3 mL/s), 3 min after a 0.25 mmol/kg prebolus of gadobutrol. MRS was performed as a 2-dimensional multivoxel chemical-shift imaging technique based on a point-resolved MRS sequence with an echo time of 135 ms over a central slice of the tumor, including contrast-enhancing parts if present. We used syngo.via (Siemens Healthcare) to semiautomatically calculate the cerebral blood volume from the perfusion raw data (including software-based leakage correction) and to assess the MRS data. For the ^18^F-FET PET, a 40-min dynamic emission recording in 3-dimensional mode consisting of 16 frames was started on injection of approximately 185 MBq of ^18^F-FET. Dynamic and static PET data were reconstructed according to our clinical protocol using a 3-dimensional ordered-subset expectation-maximization algorithm and corrections for attenuation, scatter, random events, and dead time. ^18^F-FET PET image analysis was performed as described previously and included the evaluation of both dynamic data (0–40 min after injection) and static images (summation of PET images between 20 and 40 min after injection) (*21*). ^18^F-FET tracer kinetics and maximum tumor-to-background ratio (TBR_max_) were assessed using a dedicated software package (Hermes Medical Solutions) following the current joint practice guidelines (*22*). To minimize MRI-related attenuation correction artifacts (especially for the kinetic analysis), we used a threshold-based segmentation with high thresholds, defining only the most metabolic active areas (*23*). The final interpretation of the multiparametric imaging results was produced by a board-certified neuroradiologist and nuclear medicine specialist in a clinical-routine consensus session masked to the future clinical course. Consensus reading routinely includes several measures: the presence of MRI contrast enhancement, visual hyperperfusion in dynamic susceptibility contrast perfusion MRI, a visually increased choline–to–*N*-acetyl-aspartate ratio in MRS, TBR_max_ in static ^18^F-FET PET images, and the presence of a washout curve in the kinetic PET analysis. For evaluating the accuracy of single modalities, we focused on the presence of the results mentioned above from the original PET/MRI reports.

Our patient cohort was divided into 2 main categories following the indication of ^18^F-FET PET/MRI: newly diagnosed tumors and progressive disease during or after postoperative therapy. To evaluate ^18^F-FET PET/MRI prediction metrics, we used histologic confirmation or the disease course based on follow-up examinations as ground truth. At the new diagnosis, we rated whether malignancy (World Health Organization grades III and IV) was present. At detection of progression, we defined 2 dichotomic outputs: true progression or remission. In cases with follow-up as the reference standard, malignancy or true progression was defined by the continuing imaging expansion of a tumor beginning within 3 mo after ^18^F-FET PET/MRI—the standard period until the first imaging follow-up—or by patient death within 6 mo. The absence of malignancy or progression was defined as clinically stable disease or regression without therapy for at least 6 mo. We judged other follow-up constellations as not assessable, for example, remission under continued or new therapy. Here, therapeutic effect on a vital tumor cannot be differentiated from the natural course of therapy-related changes. To estimate the impact of PET/MRI on clinical management, we tabulated the treatment changes after the disclosure of the imaging results (Table 1). We assessed whether ^18^F-FET PET/MRI was causative for a treatment change. For example, we rated ^18^F-FET PET/MRI as decisive if MRI could not determine true progression based on the Response Assessment for Neuro-Oncology criteria (*24**,**25*) but not if a treatment change was recommended before the examination but was realized only afterward.

**TABLE 1.** Categories of Clinical Management Changes Based on ^18^F-FET PET/MRI

Management change	Criteria
Active treatment to monitoring	Waiving of invasive diagnostics for tumor characterization
	Waiving of surgery or adjuvant therapy during disease course
Monitoring to active treatment	Subsequent invasive diagnostics
	Treatment start
Therapy stratification	Shift from adjuvant therapy to surgery or reversely, or change of adjuvant treatment
	Begin of or waiving additional adjuvant treatment
	Waiving planned change and continuing present treatment
Treatment adaptation	Change of location or extent of biopsy or resection
	Adjustment of irradiation volume or chemotherapy dose

### Statistical Analysis

We tabulated a confusion matrix using the respective ^18^F-FET PET/MRI single-modality outcomes and the reference standard and calculated commonly used performance metrics. Missing measurements (e.g., due to technical failure) were counted as false outcomes since they did not help solve the diagnostic question. For calculating the diagnostic performance of static ^18^F-FET PET, we used the established TBR_max_ cutoff of 2.5 at new diagnosis (*22*). At detection of progression, we performed receiver-operating-characteristics analysis for the optimal TBR_max_ cutoff, as there is no general recommendation covering a heterogeneous patient cohort. We calculated the contribution of single modalities for best predicting the reference outcome by multiple-logistic-regression analysis. We noted the percentage of cases with treatment changes based on ^18^F-FET PET/MRI results. JMP, version 15.1 (SAS), and statpages.org served as tools for the statistical calculations. In addition, we used SankeyMATIC (https://sankeymatic.com/) for building the Sankey diagrams.

## RESULTS

### Patients

**FIGURE 1.**
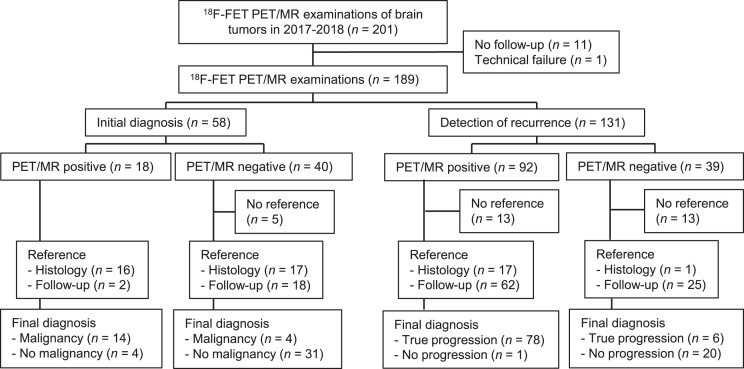
Flowchart of inclusion process for patients.

A total of 172 brain tumor patients (median age, 53 y; range, 4–86 y, 71 females) received 201 ^18^F-FET PET/MRI examinations in 2017 and 2018. Seventeen patients underwent 2, 6 patients 3, and 149 patients 1 examination. Finally, we included 189 ^18^F-FET PET/MRI examinations for evaluating the impact on clinical management and 158 for assessing the diagnostic performance (Fig. 1 provides a flow chart; Supplemental Table 1, the specific tumor pathologies; and Supplemental Table 2, the imaging characteristics of the lesions) (supplemental materials are available at http://jnm.snmjournals.org). Histologic confirmation served as the reference standard in 32% of the cases (51/158; median interval to the PET/MRI, 17 d; range, 0–113 d), and clinical and imaging follow-up served as the reference standard in 68% (107/158; median duration, 14 mo; range, 0–45 mo). Overall, ^18^F-FET PET/MRI reached an accuracy of 91% (95% CI, 85%–95%) and changed the clinical management in 47% of the cases (88/189; 95% CI, 40%–54%).

### ^18^F-FET PET/MRI at a New Diagnosis

**FIGURE 2.**
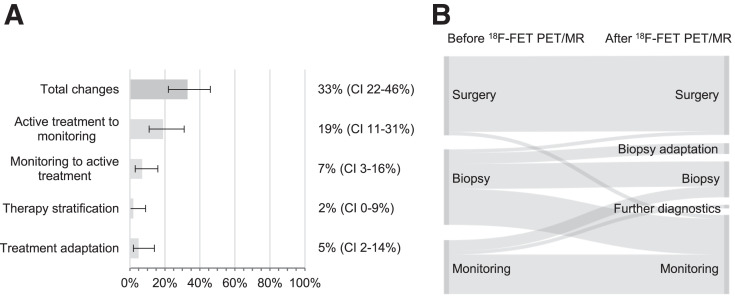
(A) Frequency (percentage with 95% CIs) of clinical management changes based on ^18^F-FET PET/MRI outcome at new tumor diagnosis. Categories are as explained in Table 1. (B) Sankey diagram showing therapies recommended before and applied after ^18^F-FET PET/MRI at new diagnosis.

The new-diagnosis category included 58 ^18^F-FET PET/MRI examinations. The indications leading to ^18^F-FET PET/MRI consisted of grading of inhomogeneous masses with predominantly low-grade features (24%, 14/58), grading in tumor locations at risk for surgery complications (14%, 8/58), identification of hot spots for biopsies (16%, 9/58), or differentiation of glioma from other entities (47%, 27/58). The accuracy of ^18^F-FET PET/MRI for identifying malignancy reached 85%, and the clinical management changed in 33% of the cases (19/58; 95% CI, 22%–46%) (Table 2; Fig. 2).

**TABLE 2.** Diagnostic Performance of ^18^F-FET PET/MRI in Clinical Setting

Parameter	New diagnosis	Detection of progression
Total case number	53	105
Disease prevalence	34%	80%
True-positive/true-negatives	14/31	78/20
False-positives/false-negatives	4/4	1/6
Sensitivity	78% (52%–94%)	93% (85%–97%)
Positive predictive value	78% (57%–90%)	99% (92%–100%)
Specificity	89% (73%–97%)	95% (76%–100%)
Negative predictive value	89% (76%–95%)	77% (61%–88%)
Accuracy	85% (72%–93%)	93% (87%–97%)

Data in parentheses are 95% CIs.

### ^18^F-FET PET/MRI at Detection of Progression

**FIGURE 3.**
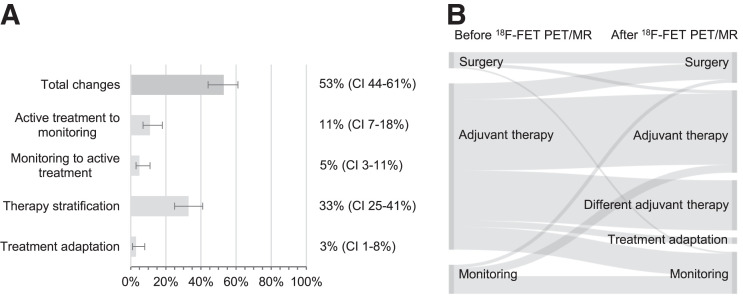
(A) Frequency (percentage with 95% CIs) of clinical management changes based on ^18^F-FET PET/MRI outcome at detection of brain tumor progression. Categories are as explained in Table 1. (B) Sankey diagram showing therapies recommended before and applied after ^18^F-FET PET/MRI at detection of progression.

In total, 131 ^18^F-FET PET/MRI examinations were performed during the disease course. The mean disease duration was 2¼ y, and 79% (104/131) of the patients had undergone surgery. The number of previously received treatments was tabulated as follows: 1 standard adjuvant therapy, either combined radiochemotherapy, radiation, or chemotherapy (27%, 36/131); 1 advanced immunotherapy or experimental treatment (2%, 3/131); 2 or more standard adjuvant therapies (15%, 19/131); standard and advanced therapy (21%, 28/131); or no adjuvant therapy within the last year (34%, 45/131). The medical history leading to ^18^F-FET PET/MRI was categorized as follows: baseline status before a new therapy (9%, 12/131), first progression under the current therapy through MRI (53%, 70/131) or with clinical symptoms (2%, 3/151), slight ongoing imaging progression (13%, 17/131), alternating imaging progression and regression (4%, 5/131), and ongoing imaging progression initially rated as therapy-associated change (18%, 24/131). ^18^F-FET PET/MRI reached an accuracy of 93% in identifying true progression and changed the clinical management in 53% (69/131, 95% CI 44%–61%) of the cases (Table 2; Fig. 3).

The largest subgroup in this category constituted 62 IDH-wild-type high-grade gliomas (anaplastic astrocytomas, World Health Organization grade III, and glioblastomas, World Health Organization grade IV). Here, the prevalence of true progression was 90%, which was detected with an accuracy of 96% (95% CI, 87%–100%) through ^18^F-FET PET/MRI. Subsequently, the clinical management changed in 47% of these patients (29/62; 95% CI, 35%–59%).

### Contribution of Single Modalities to an Optimal Disease Prediction

MRS was the modality with the most artifacts (26%, 49/189), often because of unfavorable lesion localization for the acquisition or because of measurement missing the hot-spot in large lesions (Supplemental Table 3 lists the artifacts of all modalities). At a new diagnosis, static ^18^F-FET PET and contrast enhancement yielded the highest accuracies as single modalities (83% and 79%). They also contributed most to an optimal disease prediction (*P* = 0.002 and *P* = 0.001). At detection of progression, contrast enhancement and static ^18^F-FET PET yielded the highest accuracies (80% and 79%). ^18^F-FET kinetics and static ^18^F-FET PET contributed most to an optimal disease prediction (*P* = 0.006 and *P* = 0.009; Supplemental Table 4; Table 3). Multiparametric ^18^F-FET PET/MRI using standardized criteria yielded an accuracy of 87% at new diagnosis and 89% at detection of progression.

**TABLE 3.** Contribution of Single Modalities in Multiparametric ^18^F-FET PET/MRI to Predict Outcome

	*P*	
Parameter	New diagnosis	Detection of progression
MRI contrast enhancement	0.001*	0.735
DSC MRI	0.480	0.411
MRS	0.229	0.814
Static ^18^F-FET PET	0.002*	0.009*
^18^F-FET tracer kinetics	0.939	0.006*

* Significant in multiple logistic regression.

DSC MRI = dynamic susceptibility contrast perfusion MRI.

## DISCUSSION

The accuracy of ^18^F-FET PET/MRI to identify malignancy at new diagnosis was 85%. The slightly lower sensitivity (78%) and slightly higher specificity (89%) than in a prior ^18^F-FET PET metaanalysis might be due to a more conservative interpretation of imaging findings in our study (*1*). The high specificity and negative predictive value of ^18^F-FET PET/MRI at new diagnosis might help rule out malignancy in untreated lesions. In accordance, 20% of the examined patients were able to avoid further invasive diagnostic procedures. Therefore, ^18^F-FET PET/MRI at new diagnosis may particularly benefit the significant proportion of patients with nonmalignant brain tumors, for whom a watch-and-wait strategy is sufficient. MRI contrast enhancement and static ^18^F-FET PET contributed most to the ^18^F-FET PET/MRI outcome at new diagnosis. Surprisingly, the diagnostic performance of static ^18^F-FET PET was almost as high as that of ^18^F-FET PET/MRI. Also, the proportion of clinical management changes in 33% of the patients by ^18^F-FET PET/MRI was in the range of prior reports for ^11^C-methionine PET alone, with clinical management changes in 30%–63% of the patients (*11**,**14*). Therefore, adding static amino acid PET to an existing MRI examination might be a cost-effective alternative to the multiparametric examination. Still, different studies revealed the additional value of dynamic ^18^F-FET PET for initial glioma staging, and this topic needs further evaluation (*26*–*28*).

At detection of progression, ^18^F-FET PET/MRI reached an accuracy of 93%. The prevalence of true progression, at 80%, was high per se in our cohort. Nevertheless, ^18^F-FET PET/MRI still improved the diagnostic validity. The positive predictive value reached nearly 100%, and the sensitivity (93%) and specificity (95%) were in the range of prior reports with amino acid PET/MRI (*29**,**30*). Dynamic ^18^F-FET PET was the crucial component of the multiparametric examination at detection of progression. Nevertheless, the diagnostic performance of multiparametric ^18^F-FET PET/MRI surpassed that of every single modality, and consensus reading with an individual interpretation of the results further improved the diagnostic security. ^18^F-FET PET/MRI may save time by identifying true progression in lesions with first-time imaging progression during adjuvant therapy, whereas the Response Assessment for Neuro-Oncology criteria require confirmation by follow-up MRI. This condition applied to more than half our patients at detection of progression. The prompt diagnosis accelerates effective therapy decisions, benefiting patients with a severely reduced life expectancy. Additionally, ^18^F-FET PET/MRI can clarify the nature of equivocal disease courses under therapy, another common condition in our cohort. ^18^F-FET PET/MRI changed the clinical management in 53% of the cases at detection of progression, primarily resulting in an altered therapy stratification. This proportion was slightly higher than in a previous study with ^11^C-methionine PET (*11*). On the basis of our results, the particular benefit of multiparametric ^18^F-FET PET/MRI may be the confirmation of true progression since false-positive outcomes are scarce.

The full potential of advanced MRI techniques as components of ^18^F-FET PET/MRI might not have unfolded in this study, as specialized studies reported higher accuracies (*31**,**32*). Dynamic susceptibility contrast perfusion MRI and MRS were hindered by acquisition failures and a lack of standardized quantification and might be of minor importance than amino acid PET according to our results. Future multicenter studies might explore the most efficient modality combination of ^18^F-FET PET/MRI in glioma and whether the clinical impact is higher than with a separate acquisition of the modalities (*11**,**33*). Furthermore, we did not investigate the patient outcome and cost-effectiveness directly in our study. However, it seems reasonable that waiving unnecessary invasive procedures and fast-tracking clinical management decisions are beneficial (*11**,**34*). Adding ^18^F-FET PET to MRI, such as in a hybrid scanner, has been reported to be reasonable in terms of cost-effectiveness in selected patients (*35*–*37*). Further studies considering these aspects might evaluate finally whether ^18^F-FET PET/MRI as a hybrid modality qualifies for evidence-based use in clinical routine.

Our study has several limitations. The results when performing ^18^F-FET PET/MRI examinations on clinical demand may differ from a randomized controlled trial. Our patient cohort was heterogeneous, and we did not evaluate specific histologic entities separately. The used attenuation correction for PET may have a minor impact on TBR_max_ and tracer kinetics, especially in patients with borderline findings. However, the impact can be minimized by a careful assessment of the multimodal datasets (*23*). Partially missing recommendations and clinical applications for standardized acquisition, image postprocessing, assessment, or quantification might lead to over- or underestimating the diagnostic performance of single modalities (*38*). A stepwise assessment of the single parameter’s incremental value might better identify the most efficient composition of multiparametric ^18^F-FET PET/MRI. Therefore, the exact data of this study are not generally transferable. However, it provides an exemplary insight into the actual impact of ^18^F-FET PET/MRI on clinical management of brain tumor patients outside clinical trials.

## CONCLUSION

^18^F-FET PET/MRI has high accuracy in clarifying equivocal conditions in brain tumor patients, particularly at detection of progression. The clinical value of a simultaneous examination and the optimal modality combination need further exploration. At a new diagnosis, ^18^F-FET PET/MRI appears to help rule out malignancy, with separate static ^18^F-FET PET having a comparable accuracy. During the disease course, ^18^F-FET PET/MRI facilitates clinical management by distinguishing between true tumor progression and therapy-related alterations.

## Notes

KEY POINTS **QUESTION:** Does ^18^F-FET PET/MRI improve the clinical management of brain tumor patients with equivocal findings? **PERTINENT FINDINGS:** We retrospectively evaluated the diagnostic performance of ^18^F-FET PET/MRI and its impact on clinical management at new diagnosis of brain tumors and detection of progression. ^18^F-FET PET/MRI identified malignancy or true progression with an accuracy of 91% and changed the clinical management in 47% of the cases. **IMPLICATIONS FOR PATIENT CARE:** ^18^F-FET PET/MRI as add-on diagnostics for equivocal findings in brain tumors might improve patients’ outcome by increasing the diagnostic certainty and leading to prompt changes in clinical management at different disease stages.
